# Advancing Gait Rehabilitation: A Systematic Review of Robotic Exoskeletons for Cerebral Palsy

**DOI:** 10.1017/wtc.2025.10027

**Published:** 2025-09-17

**Authors:** Amna Riaz Khawaja, Prashant K. Jamwal, Dilnoza Karibzhanova, Akim Kapsalyamov, Sunil Agrawal

**Affiliations:** 1 School of Medicine, Nazarbayev University, Astana, Kazakhstan; 2 School of Engineering and Digital Sciences, Nazarbayev University, Astana, Kazakhstan; 3 Department of Engineering and Mathematics, Hochschule Bielefeld University of Applied Sciences, Bielefeld, Germany; 4 Department of Mechanical Engineering and Department of Rehabilitation and Regenerative Medicine, Columbia University, New York, NY, USA

**Keywords:** assistive exoskeletons, biomechanics, cerebral palsy, pediatric rehabilitation, systematic review

## Abstract

Individuals with cerebral palsy (CP) experience significant impairments in lower limb mobility, which severely limit their daily activities and overall quality of life. Robotic exoskeletons have emerged as a cutting-edge solution to assist in the rehabilitation of individuals with CP by improving their motor functions. This systematic review, conducted following PRISMA guidelines, critically evaluates lower limb robotic exoskeletons specifically designed for individuals with CP, focusing on their design, rehabilitation interfaces, and clinical effectiveness. The review includes research papers published between 2010 and 2024, analyzing 30 lower limb exoskeletons reported in 57 papers. We analyze each exoskeleton, focusing on its technological features, user experience, and clinical outcomes. Notably, we identify a trend in which researchers are increasingly adapting exoskeleton functions to the specific needs of individual users, facilitating personalized rehabilitation approaches. Additionally, we highlight critical gaps in current research, such as the lack of sufficient long-term evaluations and studies assessing sustained therapeutic impacts. While ease of use remains crucial for these devices, there is a pressing need for user-friendly designs that promote prolonged engagement and adherence to therapy. This comprehensive review of existing gait rehabilitation exoskeleton technologies aimed to inform future design and application, ultimately contributing to the development of devices that better address the needs of individuals with CP and enhance their motor functions and quality of life.

## 1. Introduction

Cerebral palsy (CP) is a neuromotor disorder defined by impairments in movement, balance, and posture as a result of nonprogressive injury to the brain during the initial developmental stages of the brain (McIntyre et al., 2022; Swaroop, 2023). According to McIntyre et al. (2022), data from CP registries and population-based studies analyzing birth years from 1995 onward indicate that the birth prevalence of CP in high-income countries (HICs) ranges between 1.4 and 2.1 per 1,000 live births. Researchers have observed a downward trend in HICs, attributing it to advancements in prenatal and neonatal care, with aggregated estimates for birth after 2010 stabilizing at approximately 1.4 per 1,000 live births. While data reliability can vary, evidence suggests that CP incidence rises in low- and middle-income countries (LMICs) (McIntyre et al., 2022). Kakooza-Mwesige et al. (2017) estimated a prevalence of 2.7 per 1,000 children in Uganda, later adjusting their findings to 2.9 per 1,000 to account for attrition. Similarly, the Centers for Disease Control and Prevention (CDC) provided broader prevalence estimates ranging from 1 to nearly 4 per 1,000 live births, reflecting regional differences (C. f. D. C. a, n.d.). In contrast, earlier research, including that by Oskoui et al. (2013), reported a more universal figure of 2.1 per 1,000 live births. Collectively, the studies show that CP prevalence is higher in LMICs than in high-income nations, with a global prevalence ranging from 1.4 to 4 cases per 1,000 live births. Prenatal causes account for approximately 75% of CP cases, with perinatal asphyxia posing a significant risk factor for neonates delivered after 35 weeks of gestation (Sadowska et al., 2020).

In addition to motor deficits, individuals with CP frequently encounter disorders related to sensations, cognition, speech, behavior, and epilepsy, which may present more significant challenges than physical impairments themselves (Sewell et al., 2014). Motor impairments in CP are characterized by abnormal muscle tone, posture, and movement patterns. These results from damage in the developing brain, requiring early intervention. Notably, gait deficits represent a significant challenge, encompassing a spectrum from toe-walking to pronounced crouched gait and internal rotation of the lower limbs. Clinicians focus interventions on diagnosing and treating comorbidities like epilepsy, cognitive impairments, sensory deficits, growth, and gastrointestinal disorders, while therapists address muscle tone abnormalities through physical and occupational therapy. Rehabilitation for CP involves a collaborative approach, incorporating knowledge and skills from various fields, including physical medicine, neurology, orthopedics, rehabilitation, and assistive technology (Gulati and Sondhi, 2018). Individuals with CP face a significant challenge in rehabilitation due to elevated energy expenditure during movement. In mild cases, individuals with CP expend 32% more energy during ambulation than their typically developing peers, with energy expenditures increasing with CP severity (Bekteshi et al., 2023). Studies also show that children with CP experience lower health-related quality of life than their typically developing peers (Vila-Nova et al., 2022). Furthermore, the financial implications of care for CP exhibit considerable variation, with costs ranging from $500 to $7,500 annually in developing countries and $2,600 to $69,000 annually in developed countries. This disparity indicates the differences in healthcare accessibility and the availability of services for the CP population across various regions (Fang and Lerner, 2024).

### 1.1. Technical advancements in lower limb rehabilitation

In the past two decades, the field of rehabilitation involving robotic exoskeletons has made significant progress in retraining individuals with neurological conditions (Krebs and Volpe, 2013). According to the literature, children can begin receiving robot-assisted therapy at the age of 5–8 years (Michmizos and Krebs, 2012). Robotic exoskeletons, such as Innowalk Pro and Lokomat, have demonstrated promising outcomes for individuals with CP, improving their motor functions, gait, and overall quality of life (De Luca et al., 2022; Bonanno et al., 2023; Grodon et al., 2023). Studies also indicate that combining conventional rehabilitation with robotic assistance significantly improved outcomes in sitting, walking, and gross motor functions (Moll et al., 2022). Robotic gait training has been particularly effective, leading to improvements in walking speed, walking distance, running, and even the ability to jump (Cortés-Pérez et al., 2022). Although robotic exoskeletons yield positive outcomes in gait training, their application for individuals with CP remains challenging. This is primarily due to the complexity of the condition and the necessity for adaptive, personalized training approaches. Therapists must consider preventing overcorrection in spastic or involuntary muscle contractions when implementing robotic exoskeletons during training with children with CP. In the study by Scotto et al. (2022), researchers discussed various control strategies, emphasizing their role in promoting active motor recovery (di Luzio et al., 2022). Exoskeleton control strategies reveal that adjusting the assistance level in real-time optimizes user engagement. Several aspects still require attention before robotic exoskeletons can be routinely applied for individuals with CP, including designing and developing adaptable design and control strategies that address the specific motor impairments of individuals with CP.

### 1.2. Addressing research gaps in lower limb exoskeleton

In the past, researchers have conducted several systematic reviews to evaluate the effectiveness of lower-limb exoskeletons in improving gait performance in individuals with CP. These reviews have analyzed the literature on the current state of the art in mechanical design, actuation types, control strategies, and clinical evaluation of wearable lower limb exoskeletons, specifically for pediatric CP (Sarajchi et al., 2021). Similarly, in 2022, Hunt et al. (2022) focused on outcomes of clinical studies and possible benefits of lower-limb robotic exoskeletons to restore lower limb function.

Although these reviews offer insights, they mainly focus on certain clinical patterns and experimental conditions, restricting their relevance to the wider CP population. Furthermore, the current literature lacks thorough assessments of the long-term effects of exoskeletons on the CP population. This review addressed these deficiencies through the following comprehensive assessments:

- Clinicians and researchers evaluate the clinical efficacy of lower-limb exoskeletons in rehabilitating individuals with CP.
- Engineers and researchers advance state-of-the-art developments in design, actuation mechanisms, and control strategies.
- Investigators assess the applicability and relevance of exoskeletons for varying levels of CP severity and diverse age demographics.

This review enhances the current understanding of optimizing lower-limb exoskeletons for personalized rehabilitation in individuals with CP by combining clinical and engineering perspectives, thereby improving mobility, independence, and overall quality of life.

## 2. Classification of CP

Understanding the prognosis and selecting appropriate intervention strategies for pediatric patients with CP require clinicians to use several classifications. These classifications rely on motor types, topography, the Gross Motor Function Classification System (GMFCS), and gait patterns (McIntyre et al., 2011; Peterson and Walton, 2016). Additionally, the Manual Ability Classification System (MACS), originally developed by Eliasson et al. (2006), has been widely applied in clinical research and summarized by Paulson and Vargus-Adams (2017) to address upper extremity impairments in individuals with CP, complementing the GMFCS and other classification systems as mentioned in Paulson and Vargus-Adams (2017).

### 2.1. Motor types

This section discusses the classification of CP based on motor impairments, including spasticity, dyskinesia (encompassing dystonia and choreoathetosis), ataxia, hypotonia, and mixed types (N. I. o. N. D. a, n.d.; Dar et al., 2024).

#### 2.1.1. Spastic CP

Spastic CP is the most prevalent type among all motor types and constitutes approximately 70–80% of CP cases (Oh et al., 2019). Increased muscle tone in specific muscle groups characterizes spastic CP, causing resistance to movement in the affected extremity when a clinician applies an external force, particularly during passive stretching. This resistance intensifies with the speed of the joint movement applied and also varies with the direction of joint movement. A sudden increase in resistance at certain force levels, speeds, and angles triggers a phenomenon known as the “catch” (Skoutelis et al., 2020).

#### 2.1.2. Dyskinetic CP

Dyskinetic CP, which includes dystonia and choreoathetosis, leads to involuntary, uncontrolled movement. Damage to the basal ganglia causes either sustained movements (dystonia) or writhing/fluctuating movements (choreoathetosis), manifesting even during rest and complicating task execution. Muscles contract exaggeratedly during voluntary movements and may also activate spontaneously. Dystonia can severely impact muscle tone and posture, making it one of the most disabling forms of CP. Dyskinetic CP accounts for about 10–15% of total CP cases, following spastic CP (Monbaliu et al., 2017; Perides et al., 2020; Stewart et al., 2021).

#### 2.1.3. Ataxic CP

Cerebellar dysfunction causes ataxia, impairing coordination and balance, and accounts for 5–10% of CP cases. In contrast with dyskinetic CP, impaired motor control during voluntary movements characterizes ataxia, resulting in shaky, imprecise, and poorly executed movements. Individuals with ataxic CP struggle to keep a stable posture and also often present with intentional tremors, where these tremors get worse as they reach for an object (Sanger, 2015; Elshafey et al., 2022).

#### 2.1.4. Hypotonic CP

Hypotonic CP, a less common type affecting the entire body, accounts for 2–4% of CP cases. This condition reduces muscle tone and reflexes, significantly impairing motor functions and challenging movement, coordination, and overall physical development (Shevell et al., 2009; Sindou et al., 2020; Cooper et al., 2024).

#### 2.1.5. Mixed CP

The mixed type combines elements of spastic and dyskinetic CP, with approximately 30% of children with CP exhibiting a mixed motor pattern, demonstrating characteristics of both spastic and dyskinetic types (Termsarasab, 2017; Viswanath et al., 2023).

## 3. Topographic classification

**Figure 1.**
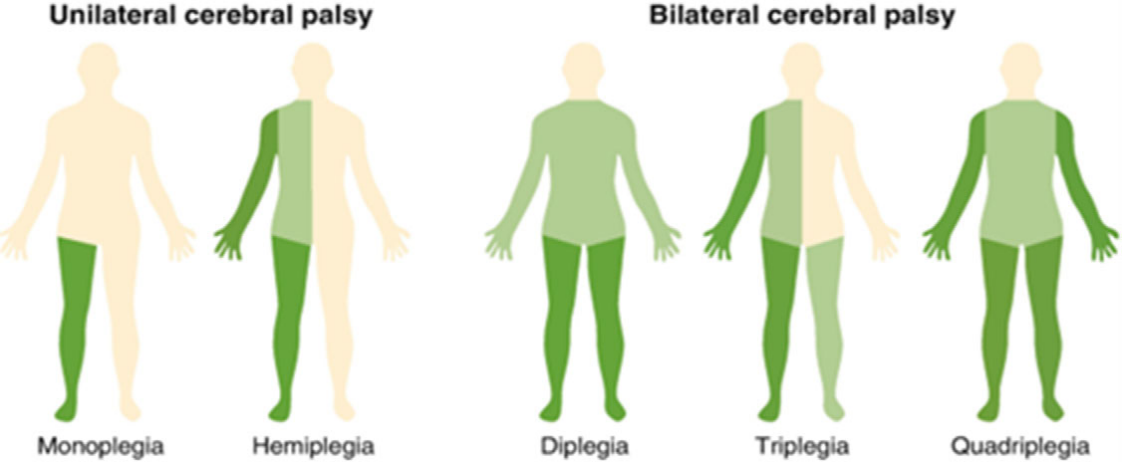
Topographic distribution of CP (Swaroop, 2023).

The topographic distribution provides a common framework for classifying CP, which is based on the distribution of motor impairment. This classification helps clinicians understand which regions of the body are affected by CP (Mandaleson et al., 2015). It can be broadly characterized as either unilateral, affecting one side of the body, or bilateral, affecting both sides (Te Velde et al., 2019). Figure 1 illustrates the topographic distribution of CP (Swaroop, 2023).

**Table 1.** Search strategy

Database	Keywords	Hits
Scopus	“Exoskeleton” OR “cerebral palsy assessment” OR “lower Limb” OR “Functional Training”	175
Web of Science	“Exoskeleton” OR “cerebral palsy assessment” OR “lower Limb” OR “Functional Training”	76
PubMed	“Exoskeleton” OR “cerebral palsy assessment” OR “lower Limb” OR “Functional Training”	106
ScienceDirect	“Exoskeleton” OR “cerebral palsy assessment” OR “lower Limb” OR “Functional Training”	128
IEEE	“Exoskeleton” OR “cerebral palsy assessment” OR “lower Limb” OR “Functional Training”	124

### 3.1. Unilateral CP

This includes monoplegia (~2–3%), affecting only one limb, either the arm or leg, and is the least common type of CP. Hemiplegia (~38–58%) affects one side of the body (arm and leg on the same side), leading to asymmetric movements, muscular weakness, and problems executing fine motor skills. Studies estimate the prevalence of unilateral CP to range from 40% to 60% (Te Velde et al., 2019).

### 3.2. Bilateral CP

Bilateral CP includes several subtypes:

- Diplegia (~30–40%) affects both lower limbs, often causing a scissoring gait.
- Triplegia (~5–7%) affects both lower limbs and one upper limb, creating asymmetrical impairment.
- Quadriplegia (~24–31%) affects all four limbs and the trunk, with more severe involvement of the upper limbs (Pakula et al., 2009; Te Velde et al., 2019).

Topographical classification is essential for identifying motor impairments in CP and helps in understanding its underlying causes. For example, research indicates a connection between spastic diplegia and premature birth, although the reliability of the data may vary. Nonetheless, evidence points to a higher prevalence of CP in individuals with low birth weight. Extensive brain damage frequently causes quadriplegia. This classification helps clinicians and therapists identify motor deficits and create targeted intervention plans (Himmelmann et al., 2021). However, topographical classification alone cannot explain the degree of functional limitation. Individuals with quadriplegia often show more severe impairments than those with hemiplegia or diplegia (Lorentzen et al., 2022).

## 4. Gross Motor Function Classification System

While topographical classification describes the distribution of motor impairments in CP, it does not explain the severity of functional limitations or mobility levels. Clinicians widely use the GMFCS to evaluate gross motor function (Leviton, 2020; Piscitelli et al., 2021). This five-level scale categorizes individuals based on their motor function, ranging from level I, which denotes the highest degree of motor function characterized by unrestricted walking, to level V, which represents the most severe limitations in motor functions, where individuals require comprehensive assistance for mobility (Compagnone et al., 2014). For further details on the GMFCS levels, readers are referred to Compagnone et al. (2014).

The GMFCS enables practitioners to classify functional abilities consistently over time, from initial diagnosis through subsequent assessments (Huroy et al., 2022). Initially, this classification system was only used for the age group 2–12 years (Piscitelli et al., 2021). Together, the GMFCS and topographical classification systems provide a comprehensive view: topography identifies the affected body regions, while the GMFCS measures functional mobility. By integrating both systems, clinicians can predict long-term outcomes, personalize rehabilitation strategies, and evaluate treatment effectiveness (Compagnone et al., 2014).

## 5. Lower-limb impairments among CP subjects

Spasticity remains a prevalent concern in individuals with CP, often leading to lower limb dysfunctions (Qin et al., 2020). Clinicians frequently observe notable impairment in the distal muscles of the lower limbs across various CP types, which compromises neuromuscular control and reduces participation in daily activities (O’Brien et al., 2020). In spastic diplegia, biomechanical abnormalities are prevalent, affecting almost all cases (98.4%) and spanning multiple regions from the pelvis to the ankle joint. These abnormalities include internal foot progression angle and internal and external rotation of the pelvis, hip, and ankle joints. Approximately 77% of children with spastic CP exhibit anomalies at multiple levels, and 48% of children exhibit anomalies at only one level (Simon et al., 2015; Zhou et al., 2017). These anomalies manifest in various forms, such as toe-walking, ankle equinus deformity, stiff knee, and scissoring gait. Torsional abnormalities, such as femoral neck anteversion and tibial torsion, are also prevalent in infantile CP and significantly impact walking patterns, increasing the risk of falls, pain, overloading, and substantial fatigue (Frizziero et al., 2022).

These biomechanical issues in the transverse plane result from a combination of static and dynamic factors, including spasticity, contractures, muscle imbalances, and excessive femoral neck anteversion. Excessive anteversion diminishes the effectiveness of hip abductors by reducing the muscular lever arms. Additionally, individuals with CP exhibit slower center-of-mass velocity at toe-off, use a wider base of support with increased step width, and have a shorter step length (Malone et al., 2016).

## 6. Methodology

### 6.1. Protocol registration and search strategy

**Figure 2.**
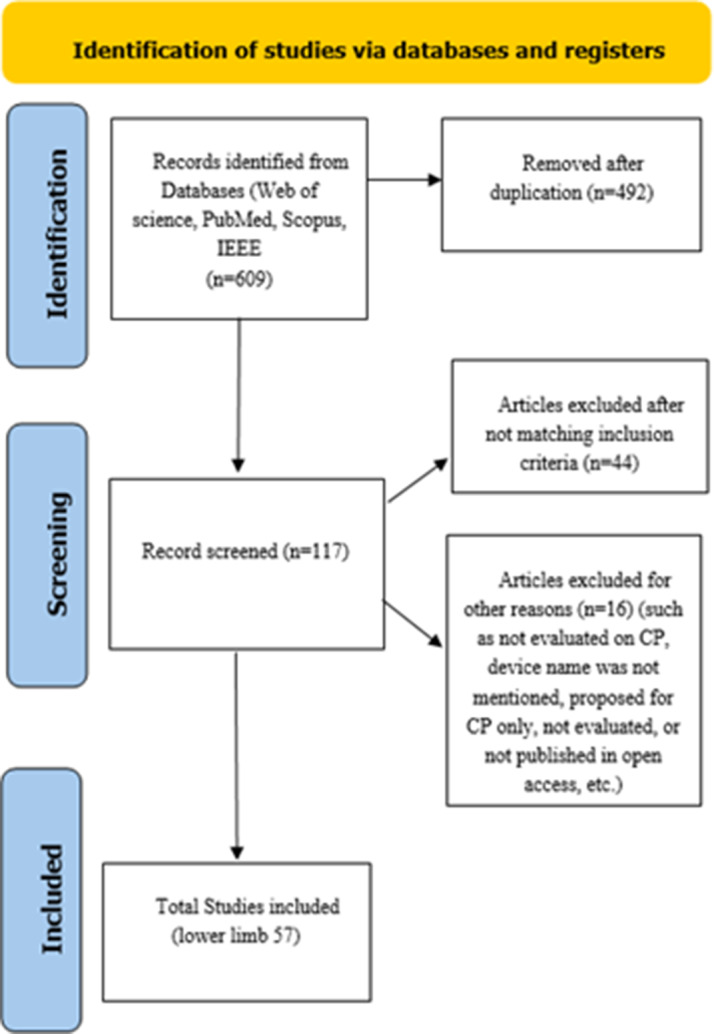
PRISMA flowchart for paper selection.

We registered this systematic review in the PROSPERO database (Registration ID: CRD42024603481) before starting data collection and analysis. This registration ensures methodological transparency and reduces potential biases in study design and reporting. We conducted the literature search on Web of Science, PubMed, IEEE Xplore, and Scopus using the keywords “exoskeletons,” “cerebral palsy,” “kinematic,” and “robotic rehabilitation.” This study particularly focused on “robot assistive gait training” and “robotic rehabilitation”. The study selection process is illustrated in Figure 2.

### 6.2. Inclusion and exclusion criteria

During this review, the articles were included based on the following criteria:

- *Population*: Studies involving children with CP.
- *Intervention*: Studies evaluating exoskeleton devices for children with CP.
- *Study design*: Any study design, including but not limited to randomized controlled trials (RCTs), cohort studies, case studies, feasibility studies, and pilot studies for exoskeletons in CP, is included to encompass a wide range of evidence.
- *Outcome measures*: Studies reporting on the design, development, or feasibility assessment of exoskeleton-based interventions for children with CP, including exoskeleton features, control algorithms, biomechanical modeling, user interfaces, and preliminary usability assessments.
- *Publication data*: Articles published in peer-reviewed journals between 2010 and 2024 to ensure relevance to current advancements in rehabilitation exoskeleton technology.

Articles were excluded based on the following criteria:

- *Population*: Studies involving individuals with neurological conditions other than CP.
- *Intervention*: Studies focusing on rehabilitation interventions other than exoskeletons, such as orthoses, therapeutic exercises, or surgical interventions, without specific emphasis on exoskeletons.
- *Study Design*: Review papers, simulation-based papers, book chapters, editorial, conference abstracts, and non-peer-reviewed articles were excluded.
- *Outcome measures*: Studies lacking relevant information on exoskeleton design, development, or feasibility assessment for children with CP.
- *Language*: Non-English articles were excluded unless deemed critical for inclusion due to limited translation resources.

## 7. Results

**Figure 3.**
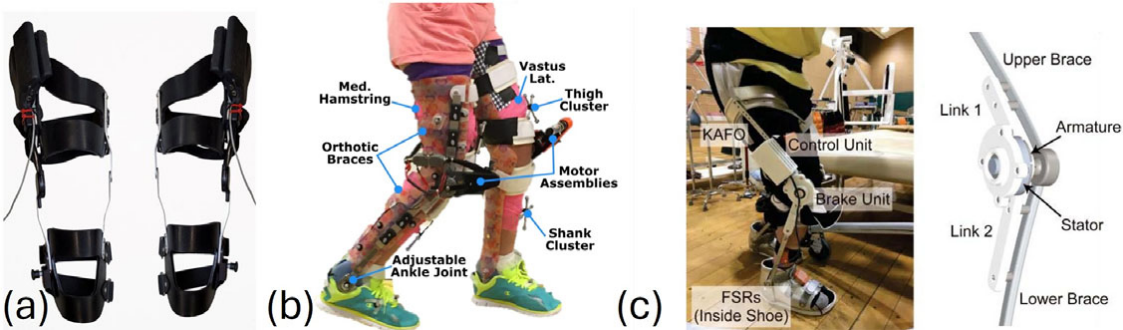
Representative knee exoskeletons designed to enhance mobility in individuals with CP: (a) bilateral knee exoskeleton (Johnson and Goldfarb, 2020), (b) tethered knee exoskeleton (Lerner et al., 2016), and (c) exoskeleton brake unit (Yamada et al., 2018). These devices vary in their actuation methods, portability, and control strategies, highlighting the evolution from passive systems to advanced, sensor-integrated designs.

**Figure 4.**
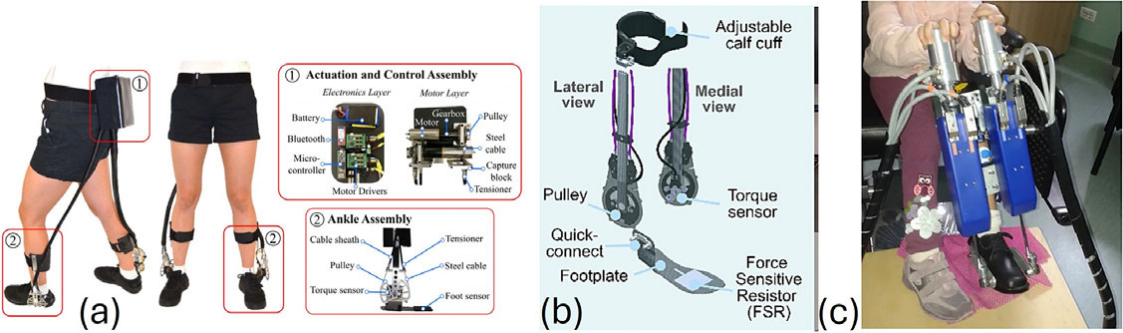
Representative ankle exoskeletons for pediatric gait training designed to enhance ankle ROM and propulsion: (a) ankle exoskeleton (Lerner et al., 2018; Lerner et al., 2019a,b; Gasparri et al., 2019; Conner et al., 2020; Orekhov et al., 2020; Conner et al., 2021; Fang et al., 2021; Fang and Lerner, 2021; Harvey et al., 2021; Fang et al., 2022), (b) ultra-light weight untethered ankle exoskeleton (Orekhov et al., 2021; Conner and Lerner, 2022; Fang and Lerner, 2022; Conner et al., 2023; Harshe et al., 2023; Fang and Lerner, 2024), and (c) PediAnklebot (Michmizos et al., 2015; Germanotta et al., 2017). These devices support gait improvement through biofeedback, real-time torque control, and gamified training.

**Figure 5.**
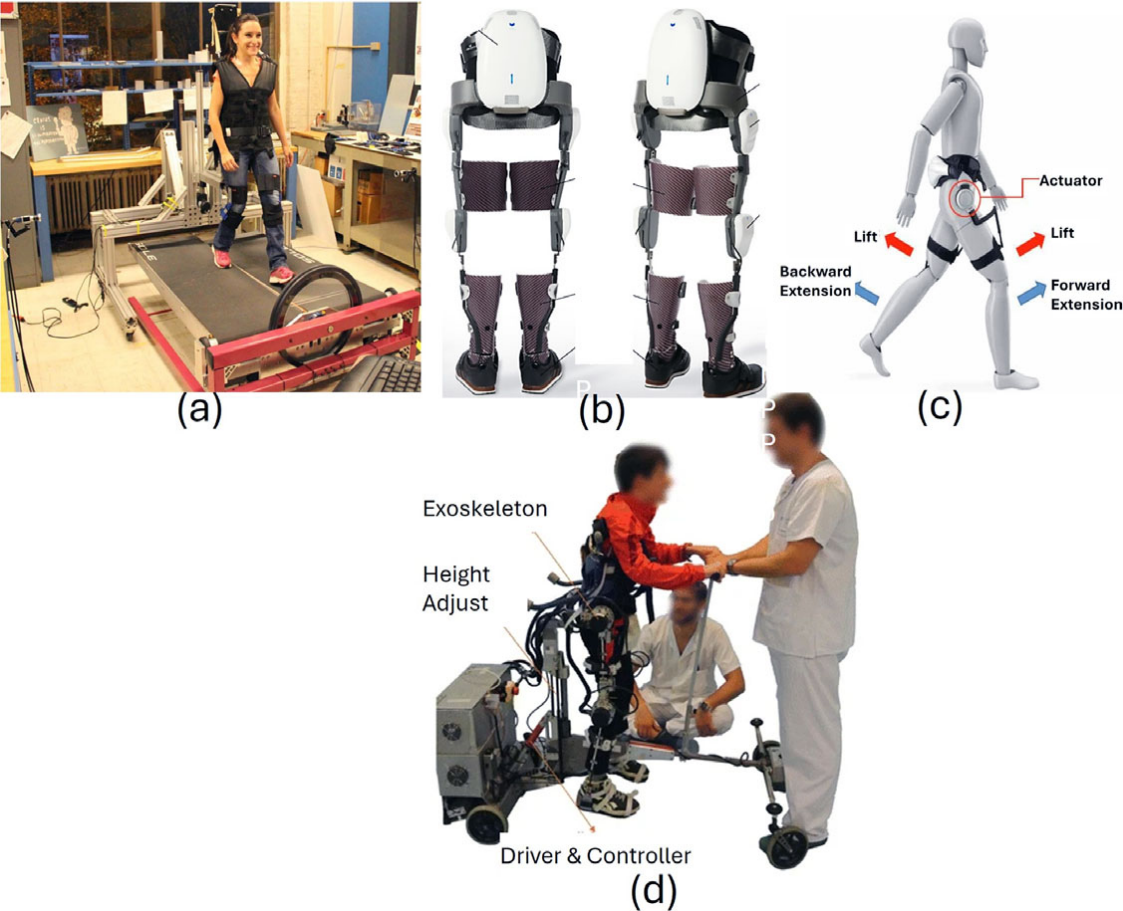
Gait exoskeletons supporting full lower limb movement: (a) MIT-Skywalker (Susko et al., 2016), (b) Angel Legs (Kim et al., 2021), (c) Honda Walking Assistant (Kawasaki et al., 2020), and (d) CP-Walker (Bayón et al., 2016a,b). These systems address walking symmetry, balance, and endurance via real-time feedback and adaptive control.

Our review revealed that exoskeleton devices for knee, ankle, and gait rehabilitation demonstrate significant trends over time in terms of weight distribution, biomechanical improvements, and functional outcomes. We included 57 studies on 30 lower-limb exoskeleton devices, which researchers clinically evaluated in the CP population. These devices, designed to support the knee, ankle, or entire lower limb for gait rehabilitation, integrate adaptive torque control, biofeedback systems, and AI-driven assistance to enhance mobility. Exoskeleton studies showed notable improvements in gait speed (up to 0.51 m/s), stride length, and knee extension (by 13.8°), while also reducing the energy expenditure for walking by up to 30%. Many devices integrate gamification and real-time feedback, allowing rehabilitation to be more engaging and effective. However, high costs, accessibility issues, and the need for long-term clinical validation remain critical challenges to overcome. Tables 2–4 detail the features, training methods, and outcomes of knee, ankle, and gait exoskeletons, respectively. Figures 3, 4, and 5 illustrate some of the knee, ankle, and gait exoskeletons that the studies reported and included in this systematic review.

**Table 2.** Knee exoskeletons: summary of engineering features, training approaches, and outcomes in rehabilitation for children with CP

Device and ref	Engineering features	Human-robot interface	Science behind training	Methods and training goals	Outcome measures and their changes	Remarks
*Tethered knee exoskeleton* (Lerner et al., 2016a)	Powered knee extension (swing and stance); PID control; custom orthotic brace with sensors	Phase-specific PID control	↓ Crouch gait; muscle normalization	6 sessions; 4 CP children (GMFCS I–III); stance, swing and combined assistance; goal: ↓ crouch gait, ↑ knee extension	↑ Knee extension 13.8° (stance); ↑ hamstring activity (some cases); partial muscle normalization	Hamstring co-activation limits benefit; longer training may improve
*Powered knee Exoskeleton* (Bulea et al., 2017)	Motorized extension assists, audiovisual gamification	Gamified audiovisual feedback	Motor Learning via gamification	6 CP children with crouched gait. Goal: enhance cortical activation, knee control.	- Knee extension: ↑ in 3/6 - EEG μ-band: ↓ indicating cortical engagement	Combines exergaming and assistance; sustained engagement noted.
*Exoskeleton brake unit* (Yamada et al., 2018)	Lightweight KAFO (~0.6 kg/leg) featuring a modular, 3D-printed electromagnetic brake unit	FRS-based real-time gait phase braking	Knee stability through flexion control	- Participants: 1 CP patient, 1 healthy individual - Assessment: Knee kinematics and muscle activity during stance and swing phases - Goal: Improve knee stability, reduce excessive flexion	- ↑ knee extension by 13.8° (stance phase, right leg) - ↑ hamstring activity in some cases (right leg) - Partial muscle activity normalization (right leg)	Enhances knee stability and support with precise gait phase control; effective for weaker legs.
*Bilateral knee Exoskeleton* (Johnson and Goldfarb, 2020)	- Powered knee module with brushless motor and chain-drive - Sensors: Encoders, IMU, FRS - Total weight: 2.0 kg	Finite-state control using FRS, IMU, and encoders for managing swing trajectories and stance torque	Biomechanical optimization.	A 63-year-old male with MAS 1+ spasticity trained in 25-meter walks with and without exoskeleton support to improve knee stability and swing kinematics	- ↓ mean knee angle by 33% (left 31% and right 35%). - ↑ peak knee flexion by 8% and total knee excursion ↑ by 52%. - ↓ walking speed by 54%, ↓ step length by 11%, ↑ step time by 62%.	Improves knee kinematics but reduces speed and stability, indicating the need for refined control strategies for both the user and the controller
*Passive knee exoskeleton* (Kennard et al., 2022)	- Supports CP patients with crouch gait. - Hydraulic disc mechanism for braking torque during stance.	Fully passive engagement based on user gait mechanics; no active control needed.	Biomechanical correction of crouch gait via repetitive passive stance phase engagement.	Participants: CP patients and healthy individuals. Training focused on improving gait stability by adjusting knee and hip joint angles and reducing excessive flexion.	↓ Hip flexion: 18.8° (left), 21.7° (right) ↓ Muscle co-activation index: Right (0.48 → 0.24), Left (0.17 → 0.017) ↑ Gmax muscle activation (right leg)	- Lightweight, simple, and cost-effective. - Requires optimization for knee support and activation timing.
*Portable pediatric knee exoskeleton* (Zhang et al., 2024)	Lightweight (1.78 kg); high torque actuators; compliant design for child growth	IMU-based deep learning (LSTM); no external feedback needed	Task-oriented control for crouch gait correction and neuromuscular training.	1 CP child (GMFCS III), 1 TDC; gait training in clinical/community settings; goal: improve stability and independence.	97% torque tracking; 94.6% gait phase estimation; ↑ compliance, gait stability; potential benefits for impaired gait.	Promising for pediatric rehab; requires further validation
*PREX* (Bulea et al., 2020; Chen et al., 2021; Bulea et al., 2022)	Single DOF at knee; custom actuator for extension assistance/resistance; FSM with real-time torque control.	FSR sensors, encoders for torque adjustment; integrated forearm crutches for stability.	Personalized torque control to improve knee biomechanics, reduce crouch gait, and lower energy cost.	Participants: 1 Pediatric CP (GMFCS III), 1 TD; gait training with alternating assistance/resistance; goal: enhance knee extension, gait, and efficiency.	↑ Knee extension, ↓ crouch gait; ↑ muscle activation (swing/stance); ↑ walking speed, ↓ metabolic cost; adaptive control superior to constant assistance.	Improves gait and motor control; safe, tolerable, and effective with forearm crutches.
*Pediatric wearable exoskeleton* (Lerner et al., 2017c)	Dynamic knee extension assistance (stance and swing); adjustable torque per user; allows overground walking	Embedded force sensors and knee encoders for real-time torque adjustment; ensures user safety.	Dynamic knee extension to recalibrate motor control and ↓ crouch gait.	Overground walking training: focus on independent ambulation and long-term benefits.	↓ Crouch gait, ↑ knee extension (midstance); ↑ muscle activity; 6/7 participants showed postural improvement.	A Promising Approach for dynamic crouch gait rehabilitation with long-term training effects.
*Modular powered exoskeleton* (Lerner et al., 2016)	Modular KAFO with powered assistance (stance and swing); lightweight and portable.	FSM with sensor feedback (force sensors) for torque regulation.	Dynamic torque reduces persistent knee flexion and improves muscle activity	Treadmill and overground walking; gradual torque adjustment for acclimation.	↑ Knee extension by 18.1°; ↑ total knee ROM by 21.0°; no ↓ in extensor activity	Safe and feasible for pediatric crouch gait rehab; long-term benefits with portability.
*Pediatric powered exoskeleton* (Lerner et al., 2017a)	Custom-molded; PID + FSM; powered stance and swing	FSM + PID based on knee angle and force	Timed extension → ↑ gait efficiency, joint	Treadmill trials; varied torque; focus: optimal stance and swing extension	↓ Knee extensor moments 35–76%; ↑ peak knee and hip extension	↓ Knee burden led to optimized biomechanics
*Powered knee exoskeleton for knee extension assistance* (Lerner et al., 2017b)	Motor/transmission for adjustable assistance torque; lacks haptic feedback	Embedded sensors for gait phase detection; torque applied	Optimized torque → improve biomechanics and ↓ crouch	Treadmill walking with progressively increased torque; data collected via motion capture, GRF, and EMG to assess knee angles and muscle activity.	↑ Torque → ↓ crouch; ↓ extensor moments; variable muscle activity	Personalization needed; positive torque–crouch link

**Table 3.** Ankle exoskeletons: summary of engineering features, training approaches, and outcomes in rehabilitation for children with CP

*PediAnklebot* (Michmizos et al., 2015; Germanotta et al., 2017)	Impedance-controlled, low-friction, back drivable; TSRT-based spasticity evaluation.	Adaptive impedance control; force sensors for real-time muscle response	Task-specific ankle training; TSRT for spasticity assessment	CP children; torque-controlled stretches at various velocities; goal: ↑ mobility, assess spasticity	↑ Ankle ROM, ↑ muscle activation, and TSRT values aligned with clinical spasticity, ↑ gait, balance, and knee ROM	Objective spasticity assessment; effective for neuro-mobility training
*Ankle exoskeleton* (Lerner et al., 2018, 2019a,b; Gasparri et al., 2019; Conner et al., 2020; Orekhov et al., 2020; Conner et al., 2021; Fang et al., 2021; Fang and Lerner, 2021; Harvey et al., 2021; Fang et al., 2022)	Lightweight, battery-powered; Bowden cable transmission; customizable torque (dorsi/plantarflexion)	Real-time torque control; step-length biofeedback	Proportional assist/resist; optimize gait and posture	CP children/adults (GMFCS I–III); multi-visit gait training across terrains; goal: ↑ walking speed, ↓ energy cost	↓ Metabolic cost (8.5–30%); ↑ walking speed (6.3–39%); ↑ step length (14–17%); ↓ EMG (12–29%); ↑ ankle power (29–44%)	Real-world viable; improves energy economy and gait stability
*Ultra-lightweight untethered ankle exoskeleton* (Orekhov et al., 2021; Conner and Lerner, 2022; Fang and Lerner, 2022; Conner et al., 2023; Harshe et al., 2023; Fang and Lerner, 2024)	Bilateral; 2.4–2.6 kg; peak torque 30 Nm; cable-driven; modular footplate and sensors	Audiovisual and EMG biofeedback; adaptive torque-based control	Resistance-based neuromuscular adaptation; ML-based prediction	CP children and unimpaired adults; incline treadmill, stairs, resistive gait; goal: ↑ flexor recruitment, ↓ compensation	↓ Soleus EMG (8–12%), gastrocnemius EMG (22%); ↓ ankle moment (12%); ↑ ankle power (66%), knee power (17%), stair ascent (38%); ↓ metabolic power (9.9–30%)	Effective for long-term mobility and ML-driven personalized training
*Adaptive ankle exoskeleton* (Bishe et al., 2021)	Same specs as (Lerner et al., 2018, 2019; Gasparri et al., 2019; Conner et al., 2020, 2021; Fang et al., 2022) terrain-adaptive proportional torque control	Seamless real-time terrain adaptation	Biomechanical adaptability across variable terrains	Validated on an incline, decline, stair ascent/descent, and 90° turn.	↓ Metabolic cost (17–28%); ↑ torque accuracy (90%), controller accuracy >87%	Scalable for real-world terrain use
*Untethered robotic ankle exoskeleton* (Gasparri et al., 2018)	Lightweight (1.996 kg); torque-based bilateral control; specs similar to (Orekhov et al., 2021; Fang and Lerner, 2022; Conner et al., 2023; Harshe et al., 2023; Fang and Lerner, 2024)	Real-time torque-based control	Ankle mechanics optimization	1 CP child (GMFCS III); treadmill training; goal: ↑ ankle power, ↓ energy cost	↑ Ankle power (45%); ↓ net metabolic rate (16%)	Enhances ankle function; supports broader clinical use
*Biomotum spark ankle exoskeleton* (Tagoe et al., 2023)	Portable, waist-mounted motor, Bowden cable ankle modules	Proportional torque via real-time ankle moment	Adaptive control for mobility gains	8 CP children (GMFCS I–III); mixed terrain training; goal: ↑ speed, ↓ energy use	↓ Metabolic cost (15–18%); ↑ walking speed (7–8%)	Improves mobility and energy use; terrain-flexible
*Wearable adaptive resistance device* (Conner et al., 2020)	Battery-powered, lightweight, bilateral (1.75 kg)	Proportional resistance control	Adaptive resistance for neuromuscular retraining	6 CP children (GMFCS I–III); 10× 20-min treadmill sessions; goal: ↑ plantar flexor strength, mobility	↑ Strength (17%); ↑ speed (39%); ↓ metabolic transport cost (33%); ↑ 6MWT (13%); ↑ TUG (11%)	Effective for strength, efficiency, and mobility rehab

**Table 4.** Gait exoskeletons: summary of engineering features, training approaches, and outcomes in rehabilitation for children with CP

*HAL 2S/ML–05* (Mataki et al., 2018; Ueno et al., 2019; Kuroda et al., 2020, 2022, 2023; Moll et al., 2022, 2023)	Hip/knee exo with CVC and CAC modes; 5 kg; bioelectric signal-based assist	EMG and GRF feedback for real-time motion correction	Motor learning, neuroplasticity, proprioceptive feedback	CP (GMFCS I–IV); 6–12 treadmill/overground sessions in 4 weeks; goal: ↑ gait speed, stride length, gross motor function	CP (GMFCS I–IV); 6–12 treadmill/overground sessions in 4 weeks; goals: ↑ gait speed, stride, GMFM. ML–05 showed no significant gains in 10MWT or 6MWT	Improves gait and motor control; ML–05 less effective on 10MWT/6MWT; standardization needed
*Lokomat® Pediatric* (Wallard et al., 2017; Wallard et al., 2018; Weinberger et al., 2019; van Kammen et al., 2020)	Motorized orthosis; BWSS; VR; adjustable actuators	Impedance control; audiovisual feedback via games	Task-specific, repetitive gait training; motor learning	– 14 CP (GMFCS II), 20 sessions (4 wks); balance/equilibrium (Wallard et al., 2018). – 3 × 12-session blocks (21 months); ↑ GMFM (GMFCS II–III) (Weinberger et al., 2019) – 20 sessions (40 min); balance and motor improvement (GMFCS II) (Wallard et al., 2017). – 10 CP (GMFCS II–III); gait trials with speed/guidance modes (van Kammen et al., 2020). Goals: ↑ dynamic balance, GMFM, motor variability	↑ speed (+0.12 m/s), ↑ step length (+0.07 m), ↓ cadence (−7.2/min), ↑ GMFM D/E (6.67–8.64%) (Wallard et al., 2018). GMFM–66 ↑6.5 pts; sustained gains (Weinberger et al., 2019). ↑ GMFM D + E by 8%, better kinematics (Wallard et al., 2017). ↓ VL/MG/TA activity, ↑ variability (~20%) (van Kammen et al., 2020)	Validated for dynamic balance and motor skills; highlights active contribution and individualized control benefits
*Lokomat® Nanos (Hocoma AG*) (Digiacomo et al., 2019)	Robotic hip/knee actuation, joint customization	Cooperative impedance control with VR feedback	Neuroplasticity-driven gait cycle repetition	CP, SCI, stroke, MS; treadmill-based training; goal: ↑ gait function, endurance, voluntary effort	- ↑ Walking speed - ↑ Gait endurance, - ↑ Voluntary muscle effort - ↓ Spasticity and better gait symmetry	Feasible for severe impairments; needs validation for balance and overground.
*CP walker* Bayón et al., 2016a,b)	Smart walker with active hip/knee/ankle joints	Multimodal (EEG/EMG/IMU) interface; AAN control	CNS-PNS integrated feedback for neurorehabilitation	CP (GMFCS II–IV); 10 adaptive sessions over 5 weeks; goal: ↑ gait symmetry, stability, and weight-bearing. Tailored therapies using biofeedback and Assist-as-needed (AAN) strategies	Improved gait symmetry, velocity (+51.94%), cadence (+29.19%), step length (+26.49%), and weight-bearing capacity	Promising for CP rehabilitation but requires validation in larger clinical trials.
*WAKE-Up exoskeleton* (Patané et al., 2017)	Modular KAFO with SEA (Series Elastic Actuators); 2.5 kg	Gait-phase-based position control	Biomechanical torque support for joints	3 hCP trials under 3 walking conditions; goal: restore physiological gait	↑ knee flexion, ↑ ankle dorsiflexion (swing), ↑ hip extension (pre-toe-off)	Supports gait improvement; refines knee module
*MIT-Skywalker* (Susko et al., 2016)	Split-belt treadmill with adaptive BWSS	Real-time adaptive control and Visual feedback for balance/gait training	Patient-driven motor learning with feedback	CP/stroke; 3–4 sessions/week for 1 month; goal: ↑ gait symmetry, balance, walking speed	↑ speed (+30%), ↑ balance (BBS +270%), ↓ asymmetry (11% → 2%)	Versatile; suitable for expansion to larger studies
*Honda walking assistance* (Kawasaki et al., 2020)	Lightweight, Wearable hip exo with bilateral actuators	Torque via potentiometers; real-time symmetry assist	Bilateral motor learning focus	CP; sham-controlled treadmill trial; goal: ↑ gait symmetry and propulsion	↑ Hip ROM (+19% flexion, +27% extension), ↑ limb symmetry (+13%); ↑ propulsion force (affected limb)	Effective for short-term gait symmetry and propulsion
*ProGait* (McDaid, 2017)	Robotic frame with hip/knee ROM control	Force-field single-point guidance	Adaptive gait support for crouch gait	CP (GMFCS II–IV); overground therapy; goal: normalize gait, ↑ activation	↓ knee flexion, ↑ hip/knee ROM, ↑ joint torque	Innovative home-use potential; needs more validation
*ATLAS2030* (Delgado et al., 2021)	8-joint pediatric exoskeleton (hip/knee/ankle), stiffness adjustable	Automatic and active modes with safety settings	Repetitive gait and strength therapy	CP (GMFCS III–IV); 10 sessions/month; goal: ↑ ROM, ↓ spasticity, ↑ strength	↑ ROM: hip (+21.5°), knee (+6.5°), ankle (+2.5°); ↑ strength (+55 N); ↓ spasticity	Safe for moderate-to-severe CP; larger trials needed
*Passive Pediatric Leg Exoskeleton* (Zistatsis et al., 2021)	Lightweight passive springs for gait assist	Fully passive, no interface	Mechanical energy return	CP and non-CP; walk trials; goal: ↓ energy cost, ↑ joint mobility	↓ energy cost (8%), ↑ joint kinematics	Promising low-cost tool; refinement needed
*Angel Legs* (Kim et al., 2021)	Hip/knee exo with 4 actuated joints; ground sensors	Dynamic torque with impedance reduction	Motor learning via assisted motion	CP (GMFCS II–IV); 17–20 overground sessions; goal: ↑ distance, endurance, motor skill	↑ GMFM (up to 10%), ↑ 6MWT (+140 m), ↓ O₂ cost (37–75%)	Good for moderate/severe CP; endurance benefit
*EksoGT* (Manikowska et al., 2021)	VR-integrated treadmill exo with adjustable support	Biofeedback for gait symmetry	Gait/balance motor learning	CP: 10-week program (30 sessions); goal: ↑ symmetry, balance	↑ Symmetry in support/walking speed	No major gait parameter changes; small sample size

Tables 2–4 Features, training methods, and outcomes of lower limb exoskeletons used in rehabilitation for individuals with cerebral palsy (CP). Table 2 details knee exoskeletons, focusing on improvements in knee extension and gait stability. Table 3 covers ankle exoskeletons, emphasizing enhancements in plantarflexion and agility. Table 4 addresses gait exoskeletons supporting the entire lower limb, highlighting improvements in gait speed and energy expenditure. Note: Some devices (e.g., untethered ankle exoskeleton, Biomotum Spark) may appear in multiple tables due to their relevance to ankle or gait rehabilitation applications.

**Table 5.** Summary of exoskeleton classes across key design and clinical metrics

Device category	Typical weight range	Common actuator types	Common control strategies
Knee exoskeleton	~3–7 kg	Electric motors, pneumatics	PID, adaptive torque, FSM
Ankle exoskeleton	~1.5–4 kg	Electric motors, SEAs, pneumatic artificial muscles	Proportional joint moment, biofeedback-based,
Gait (full lower limb)	~10–30 kg (Lokomat >100 kg)	Electric motors, hydraulic, series elastic actuators	Assist-as-needed, impedance control, ML-Based, FSM, VR Feedback

Tables 2–4. Features, training methods, and outcomes of lower limb exoskeletons used in rehabilitation for individuals with cerebral palsy (CP). Table 2 details knee exoskeletons, focusing on improvements in knee extension and gait stability. Table 3 covers ankle exoskeletons, emphasizing enhancements in plantarflexion and agility. Table 4 addresses gait exoskeletons supporting the entire lower limb, highlighting improvements in gait speed and energy expenditure. Note: Some devices (e.g., untethered ankle exoskeleton, Biomotum Spark) may appear in multiple tables due to their relevance to ankle or gait rehabilitation applications.

**Figure 6.**
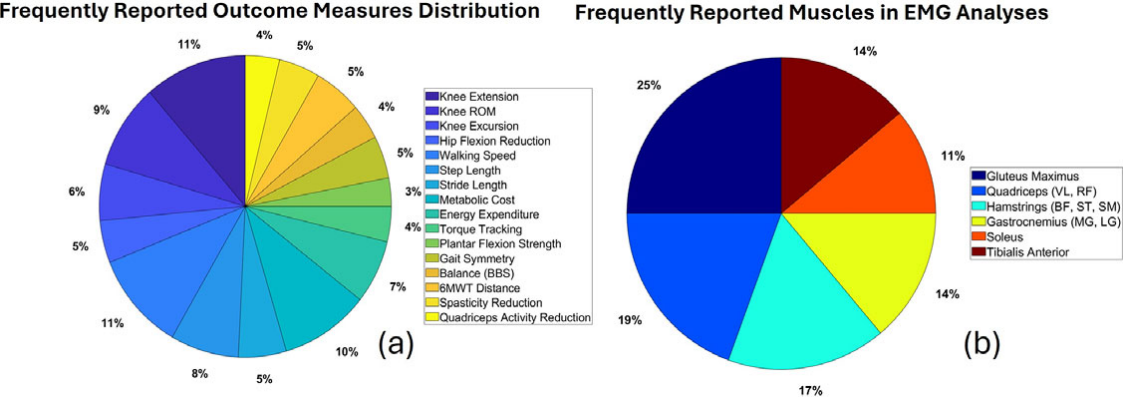
Outcome measures and muscle groups assessed in studies: (a) commonly reported outcomes in CP exoskeleton studies include knee extension, crouch gait reduction, gait stability, and cortical activation. (b) Frequently analyzed muscle groups via EMG include gluteus maximus, quadriceps, hamstrings, gastrocnemius, soleus, and tibialis anterior, highlighting a focus on muscles critical to gait propulsion and postural stability.

Figure 6(a) illustrates the frequently reported outcomes and highlights the functional measures used to assess exoskeleton effectiveness. Knee extension, reported in 25% of studies, is a critical outcome for improving gait function. Reduction in crouch gait, reported in 18% of studies, is a key outcome for individuals with CP, following knee extension in frequency. Other outcomes include gait stability (10%), cortical activation (5%), and torque and gait adaptation (5%). These findings indicate the need for a multifaceted approach to assess lower limb exoskeleton performance, incorporating mechanical and neurological outcomes.

Figure 6(b) illustrates the frequency of muscle groups analysis in studies involving individuals with CP. Figure 6(b) presents the data in six distinct categories, each assigned unique colors and percentages to aid interpretation. The gluteus maximus was analyzed in 25% of the studies, which is the most frequently reported muscle. This reflects its critical role in hip extension and stability during locomotion, key aspects of gait mechanics. The quadriceps, including the vastus lateralis and rectus femoris, account for 19% of analyses, reflecting their critical role in knee extension and lower limb function. The hamstrings (biceps femoris, semitendinosus, and semimembranosus) account for 17% of analyses, whereas the gastrocnemius (medial and lateral heads) represent 14%. Both muscle groups play essential roles in knee flexion and ankle plantarflexion, respectively. The soleus (14%) and tibialis anterior (11%) are less frequently analyzed. The soleus plays a critical role in plantarflexion, while the tibialis anterior is integral to dorsiflexion. This may reflect challenges in examining these muscles or focusing on more proximal muscle groups. Overall, the data highlight a stronger emphasis on the gluteus maximus and quadriceps due to their prominent roles in locomotor stability and the high prevalence of gait abnormalities among individuals with CP. This distribution likely reflects the need to analyze muscles most critical to walking mechanics and mobility impairment.

**Figure 7.**
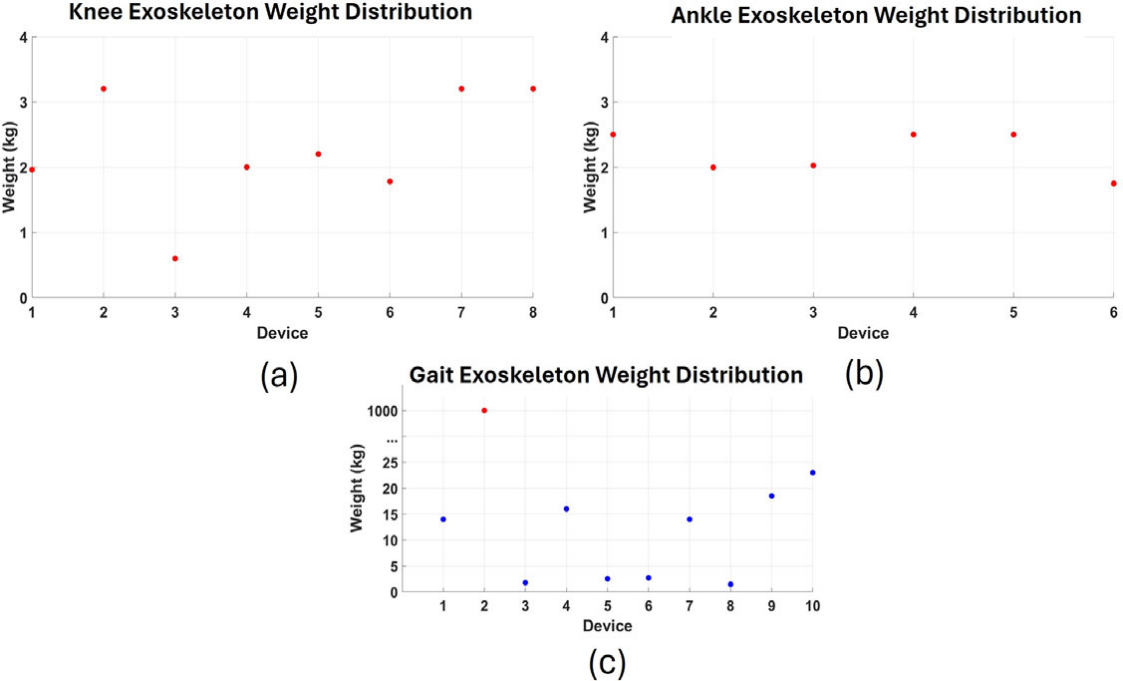
Weight distributions in knee, ankle, and gait exoskeletons: (a) Scatter plot of the weight distribution of knee exoskeletons included in this review. Devices: 1) tethered knee exoskeleton (1.96 kg), 2) powered knee exoskeleton (3.2 kg), 3) exoskeleton brake unit (0.6 kg), 4) bilateral knee exoskeleton (2.0 kg), 5) passive knee exoskeleton (2.2 kg), 6) portable pediatric knee exoskeleton (1.78 kg), 7) PREX (3.2 kg), and 8) pediatric modular/powered exoskeleton (3.2 kg). (b) Scatter plot showing the weight distribution of ankle exoskeletons included in this review. Devices: 1) Biomotum spark ankle exoskeleton (2.4–2.6 kg), 2) untethered robotic ankle exoskeleton (1.996 kg), 3) adaptive ankle exoskeleton (1.85–2.2 kg), 4) ultra-lightweight untethered ankle exoskeleton (2.4–2.6 kg), 5) PediAnklebot (2.5 kg), and 6) wearable adaptive resistance device (1.75 kg). (c) Scatter plot showing the weight distribution of gait exoskeletons included in this review. Devices: 1) hybrid assistive limb (1.76–14 kg), 2) CP Walker (14–18 kg), 3) WAKE-Up exoskeleton (2.5 kg), 4) Honda Walking Assistance (2.7 kg), 5) ATLAS2030 (14 kg), 6) passive pediatric leg exoskeleton (1.45 kg), 7) angel legs (18.5 kg), 8) EksoGT (23 kg). Note: Lokomat® Pediatric (>1,000 kg) is excluded from the plot due to its extreme weight.

Figure 7a–c illustrate the weight distribution of knee, ankle, and gait exoskeletons, respectively. The scatter plots highlight substantial variation across device categories. Ankle exoskeletons are the lightest, typically weighing under 3 kg, supporting agility and user comfort. Knee exoskeletons display a moderate weight range (approximately 0.6–3.2 kg), balancing portability and support. Gait exoskeletons exhibit the greatest variability, with most devices falling between 2 and 23 kg. Notably, Lokomat® Pediatric exceeds 1,000 kg and was excluded from the plot due to its outlier status. These weight differences underscore a key trade-off between functional assistance and usability in real-world environments.

**Figure 8.**
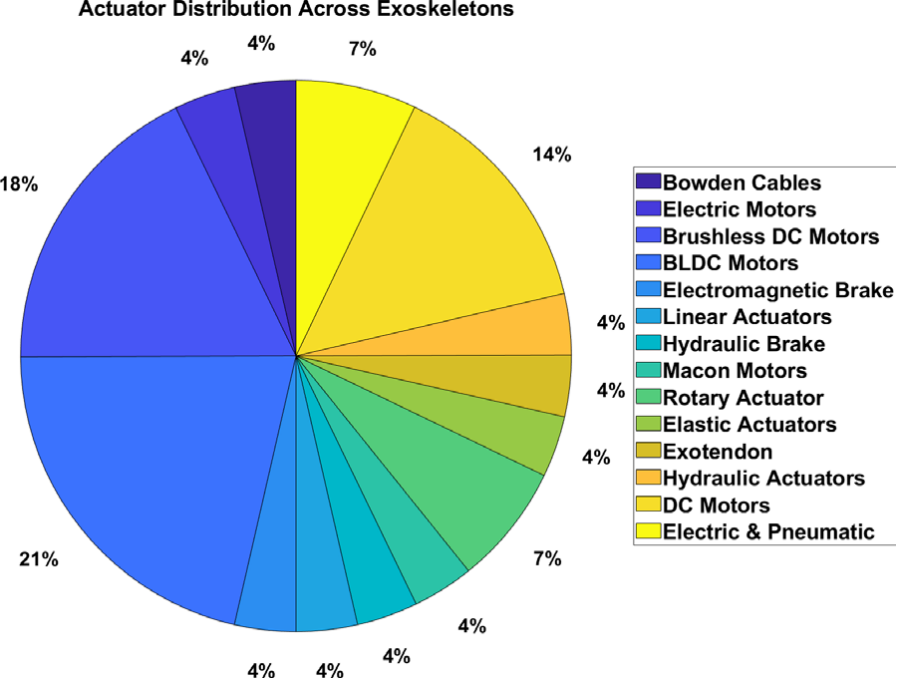
Frequency distribution of actuator types in lower-limb exoskeletons for individuals with CP: This pie chart illustrates the distribution of actuator types utilized in lower limb exoskeletons designed for individuals with CP. Electric motors are the most prevalent, followed by hydraulic, pneumatic, and series elastic actuators. The frequencies of actuator use are represented as percentages.

Based on the analysis of actuators used in lower-limb exoskeletons, shown in Figure 8, electric motors dominate as the most frequently employed actuator type due to their energy efficiency, precision, and flexibility. In contrast, hydraulic and pneumatic actuators are more powerful but generally heavier and less energy efficient, making them less appropriate for portable rehabilitation devices. Common pathological gait patterns in CP include muscle activation (15%), step length and speed (12%), and metabolic cost (10%).

Additionally, series elastic actuators possess improved compliance and safety, with the ability to modulate forces, but possess a less responsive performance compared to direct-drive actuators. The findings demonstrate a continuous effort to optimize actuators, balancing efficiency, power delivery, and user comfort. This constitutes a big area of innovation in lower limb exoskeleton design. For reference, all acronyms used throughout this review are summarized in Table 6, and the notations of symbols (e.g.,↑, ↓, %) used on reporting results summarized in Table 7.

**Table 6.** Acronyms and their abbreviations

Acronyms	Abbreviations
AAN	Assist as-needed
ABI	Acquired brain injury
BWS	Body weight support
BWSS	Body weight support system
CAC	Cybernic autonomous control
CDC	Center for Disease Control and Prevention
CNS	Central nervous system
COM	Center of mass
COP	Center of pressure
CP	Cerebral palsy
CVC	Cybernic voluntary control
DOF	Degree of freedom
EEG	Electroencephalography
EMG	Electromyography
FSR	Force-sensitive resistor
FSM	Finite state machine
GMFCS	Gross Motor Function Classification System
GMFM	Gross motor function measure
GRF	Ground reaction force
hCP	Hemiplegic cerebral palsy
HAL	Hybrid assistive limb
HICs	High-income countries
IMU	Inertial measurement unit
KAFO	Knee ankle foot orthosis
LMICs	Low- to middle-income countries
LSTM	Long short-term memory (AI model for gait prediction)
MAS	Modified Ashworth Scale (measures spasticity)
MG	Medial gastrocnemius
ML	Machine learning
MoS	Margin of stability
NAGT	Non-robotic assisted gait training
PCI	Physiological cost index
PID	Proportional-integral-derivative control
PNS	Peripheral nervous system
PREX	Pediatric robotic exoskeleton
PRISMA	Preferred Reported Items for Systematic Reviews and Meta-Analyses
PROSPERO	International Prospective Register of Systematic Reviews
RAGT	Robot-assisted gait training
RCT	Randomized controlled trials
RoM	Range of motion
sCP	Spastic cerebral palsy
SCI	Spinal cord injury
TDC/TD	Typically developing child/children
TA	Tibialis anterior
TSRT	Tonic stretch reflex threshold
TUG/TUGT	Timed up and go test
VL	Vastus lateralis
VR	Virtual reality

**Table 7.** Symbols and their meanings

Symbols	Meaning
↑	Increase
↓	Decrease
±	Plus/minus (use for standard deviation)
≥	Greater than or equal to
≤	Less than or equal to
~	Approximately
→	Leads to/results in
°	Degree
m/s	Meters per second
m	Meters
Kg	Kilograms
Nm	Newton-meters
%	Percentage

This comprehensive analysis of weight allocation, biomechanical improvements, outcome measures, and actuator design offers significant insight into the current performance and limitations of lower limb exoskeletons, highlighting the need for further research and technological advancements in the field.

## 8. Discussion

CP affects around 17 million people worldwide and is characterized by motor disorders, involving motor disorders that reduce mobility (Navarro et al., 2024). Robot-assisted rehabilitation is designed to contribute to improvements in motor function, gait efficiency, and enhancing functional outcomes, which would ultimately lead to physical independence in the CP population. Improvement in function as a result of robotic rehabilitation is based on neuroplasticity and motor learning, which results from repetitive motion aimed at modifying and reorganizing neuronal connections and networks within the central nervous system (CNS) (Lim et al., 2024).

Engineers have designed robotic exoskeletons to deliver controlled and repetitive movement with targeted assistance following the principles of motor learning and neuroplasticity. The devices reviewed in this work focused on promoting active participation through targeted therapy, addressing specific impairments in the lower limbs as well as overall gait mechanics. While integrating advancements in robotics, biofeedback, and adaptive control systems, these devices have become critical tools in CP rehabilitation. However, significant challenges remain, including cost, accessibility, and the need for longer-term efficacy studies. This discussion explores advancements in exoskeleton design, emerging trends, evaluating the efficacy of training methodologies, analyzing their strengths and challenges, and implications for rehabilitation. The discussion emphasizes modular and adaptive designs while also contemplating future directions in this rapidly evolving field.

### 8.1. Evolution of exoskeleton design

In the last decade, exoskeletons have undergone substantial advancements in their design while transitioning from rigid to more adaptive and flexible configurations. Previous models like Lokomat (Wallard et al., 2017, 2018; Digiacomo et al., 2019; Weinberger et al., 2019; van Kammen et al., 2020) and EksoGT (Manikowska et al., 2021) predominantly employed rigid metallic structures to provide support and facilitate movement. Recent exoskeleton designs integrate lightweight materials, adaptive control strategies, and real-time biofeedback through VR and gamification interfaces, enhancing patient engagement and rehabilitation outcomes (Lerner et al., 2016; Lerner et al., 2017a,b; Bulea et al., 2017; Gasparri et al., 2018; Orekhov et al., 2021; Conner and Lerner, 2022; Fang and Lerner, 2022; Conner et al., 2023; Harshe et al., 2023; Tagoe et al., 2023; Fang and Lerner, 2024). Earlier knee designs, as seen in the tethered knee exoskeleton (Lerner et al., 2016), primarily focused on powered assistance with simple PID control strategies. Over time, studies have incorporated advanced sensor technologies, including force sensitive resistors (FSRs), inertial measurement units (IMUs), and encoders, to optimize the control mechanism. (Bayón et al., 2016a,b; Bulea et al., 2020; Johnson and Goldfarb, 2020; Chen et al., 2021; Bulea et al., 2022; Zhang et al., 2024). Exoskeletons such as ATLAS2030 (Delgado et al., 2021), Hybrid Assistive Limb (HAL) (Mataki et al., 2018; Ueno et al., 2019; Kuroda et al., 2020, 2022, 2023; Moll et al., 2022, 2023), Angel Legs (Kim et al., 2021), and CP Walker (Bayón et al., 2016a,b) include more degrees of freedom to improve movement flexibility and meet various user requirements.

Exoskeletons have also progressed from being confined to laboratory settings to actual real-world settings or are feasible to be adapted to real-world settings (Lerner et al., 2018; Lerner et al., 2019a,b; Gasparri et al., 2019; Conner et al., 2020; Orekhov et al., 2020; Conner et al., 2021; Fang et al., 2021; Fang and Lerner, 2021; Harvey et al., 2021; Fang et al., 2022).

Exoskeletons such as ATLAS2030 (Delgado et al., 2021) (a pediatric gait exoskeleton), Biomotum Spark (Tagoe et al., 2023) (an ankle exoskeleton with biofeedback for gait correction), Lokomat® (Wallard et al., 2017, 2018, Digiacomo et al., 2019, Weinberger et al., 2019, van Kammen et al., 2020) (a treadmill-based gait trainer), CP Walker (Bayón et al., 2016a,b) (a gait rehabilitation device), HAL (Michmizos et al., 2015; Lerner et al., 2016; Susko et al., 2016; Johnson and Goldfarb, 2020; Kim et al., 2021; Conner and Lerner, 2022; Swaroop, 2023), Ekso GT (Manikowska et al., 2021), MIT-Skywalker (Susko et al., 2016), Angel Legs (Kim et al., 2021), and ProGait (McDaid, 2017) emphasize portability and usability in everyday environments. A comparative overview of knee, ankle, and gait exoskeletons across the weight, actuators, and control dimensions is presented in Table 5.

In the assessment of exoskeleton design, numerous critical factors influence both performance and usability.

#### 8.1.1. Energy efficiency

The design of contemporary exoskeletons has been enhanced through the optimization of battery life and the reduction of power consumption, as demonstrated by autonomous systems such as Biomotum Spark (Tagoe et al., 2023), Angel Legs (Kim et al., 2021), and Honda Walking Assistance (HWA) (Kawasaki et al., 2020).

#### 8.1.2. Biomechanical adaptability

It is a focal point in these devices. For example, ProGait (McDaid, 2017), HAL (Mataki et al., 2018; Ueno et al., 2019; Kuroda et al., 2020, 2022, 2023; ; Moll et al., 2022, 2023), HWA (Kawasaki et al., 2020) prioritize adherence to normal gait patterns and improves hip symmetry during ambulation.

#### 8.1.3. User comfort and ergonomics

Designers have considered that weight distribution, soft padding, and the ability to adjust the fit are critical considerations, as demonstrated in CP Walker (Bayón et al., 2016a,b), Angel Legs (Kim et al., 2021), and HWA (Kawasaki et al., 2020).

#### 8.1.4. User adherence and practicality

Despite significant progress in exoskeleton technology for CP, aspects such as user acceptance, long-term adherence, and usability remain underexplored in the current literature. Most studies have prioritized mechanical design, biomechanical outcomes, and short-term clinical improvements, while neglecting sustained user engagement. For instance, works by Lerner et al., Fang and Lerner, and Gasparri et al. incorporate promising features such as audiovisual gamification and biofeedback, such as in the powered knee exoskeleton (Lerner et al., 2016, 2017a,b; Bulea et al., 2017), ankle exoskeletons (Lerner et al., 2018, 2019a,b; Gasparri et al., 2019; Conner et al., 2020, 2021; Orekhov et al., 2020; Fang et al., 2021, 2022; Fang and Lerner, 2021; Harvey et al., 2021) that can enhance user motivation. However, large-scale evaluations of user satisfaction, dropout rates, or long-term use are notably absent. Some studies suggest that factors such as ease of setup, comfort, and interactive features contribute positively to user engagement, as seen in the powered knee exoskeleton (Lerner et al., 2016, 2017a,b; Bulea et al., 2017), yet psychosocial aspects and real-world usability remain insufficiently documented. This highlights a critical limitation in the field. Future research should systematically assess user-centered outcomes to ensure that exoskeletons are not only effective but also practical and acceptable in everyday rehabilitation settings.

#### 8.1.5. Sensor integration and feedback mechanisms

Devices such as PediAnklebot (Michmizos et al., 2015; Germanotta et al., 2017), CP Walker (Bayón et al., 2016a,b), and HWA (Kawasaki et al., 2020) offer real-time monitoring and adaptive assistance. The latter utilizes potentiometer-based torque control.

#### 8.1.6. Modularity and scalability

ATLAS2030 (Delgado et al., 2021) is adaptable to different age groups and severity levels.

#### 8.1.7. Actuation mechanism

The transition from big electric actuators to more power-efficient mechanisms, that is, series elastic actuators (SEAs) in HAL (Mataki et al., 2018; Ueno et al., 2019; Kuroda et al., 2020, 2022, 2023; Moll et al., 2022, 2023) and small brushless DC motors in different devices, for example, ProGait (McDaid, 2017), Biomotum Spark (Tagoe et al., 2023), bilateral knee exoskeleton (Johnson and Goldfarb, 2020), and untethered ankle exoskeleton (Gasparri et al., 2018; Orekhov et al., 2021; Conner and Lerner, 2022; Fang and Lerner, 2022; Conner et al., 2023; Harshe et al., 2023; Fang and Lerner, 2024), has improved response time and power efficiency.

#### 8.1.8. Control strategies

Exoskeletons have progressed from basic preprogrammed assistance to advanced and adaptive control systems. Exoskeletons like the HAL Mataki et al., 2018; Ueno et al., 2019; Kuroda et al., 2020, 2022, 2023; Moll et al., 2022, 2023) and Angel Legs (Kim et al., 2021) utilize impedance and assist-as-needed control mechanisms, while PediAnklebot (Michmizos et al., 2015; Germanotta et al., 2017) and CP Walker (Bayón et al., 2016a,b) incorporate machine learning-based adaptation for real-time gait modification.

Furthermore, the ProGait (McDaid, 2017) and WAKE-Up (Patané et al., 2017) Exoskeletons utilize finite-state machine control to improve user responsiveness and maximize energy efficiency. Knee exoskeletons like PREX (Bulea et al., 2020; Chen et al., 2021; Bulea et al., 2022) and powered knee exoskeleton (Lerner et al., 2016, 2017a,b; Bulea et al., 2017) utilize PID and adaptive torque control mechanisms, while ankle exoskeletons such as Biomotum Spark (Tagoe et al., 2023) and untethered ankle exoskeleton (Gasparri et al., 2018; Orekhov et al., 2021; Conner and Lerner, 2022; Fang and Lerner, 2022; Conner et al., 2023; Harshe et al., 2023; Fang and Lerner, 2024) employ proportional joint moment control in conjunction with biofeedback-based adaptation to enhance gait correction. The HWA device utilizes potentiometer-based torque control to deliver real-time bilateral hip support, thereby improving propulsion and limb symmetry.

## 9. Study limitations and contradictory findings

Although lower-limb exoskeletons exhibit significant potential for improving gait and motor function in children with CP, multiple studies present contradictory results and limitations that warrant attention. As reported in the study (Yamada et al., 2018), the stability of the knee joint was only improved in the supported limb, indicating a limitation in attaining bilateral improvements. The tethered knee exoskeleton (Lerner et al., 2016) exhibited increased hamstring activity in some cases, which occasionally reduced kinematic improvements, reflecting heterogeneity in muscular responses among individuals. Several studies, including the assessments of the Exoskeleton Brake Unit (Yamada et al., 2018) and preliminary PediAnklebot (Michmizos et al., 2015; Germanotta et al., 2017) consist of small sample sizes (e.g., individual patients or small cohorts), limiting their generalizability to diverse CP populations.

The lack of long-term follow-up in the majority of studies limits their understanding of lasting treatment effects. Differences in outcome measurements (such as gait velocity compared to muscle activation) and efficacy among CP classifications (including spastic versus dyskinetic) or GMFCS levels complicate comparisons. These differences highlight the critical need for standardized methodologies, comprehensive multicenter studies, and longitudinal investigations to assess the efficacy of exoskeletons and to tackle the variability in outcomes.

## 10. Cost and accessibility challenges in LMICs

The cost and accessibility of robotic exoskeletons are critical issues, especially for the global CP population. As mentioned previously, considerable annual cost disparities for CP care range from $500 to $7500 in LMICs to $2,600 to $69,000 in HICs (Fang and Lerner, 2024). These figures reflect substantial differences not only in healthcare expenditure but also in the accessibility of advanced rehabilitation technologies.

The high production and maintenance costs of commercial exoskeletons, as well as the need for specialized setup and training facilities such as Lokomat® (Wallard et al., 2017, 2018; Weinberger et al., 2019; van Kammen et al., 2020), HAL (Mataki et al., 2018; Ueno et al., 2019; Kuroda et al., 2020, 2022, 2023; Moll et al., 2022, 2023), pose significant barriers to their widespread adoption in resource-limited settings. While more affordable and passive devices, such as the passive knee exoskeleton (Kennard et al., 2022), passive pediatric leg exoskeleton (Zistatsis et al., 2021) offers promise in terms of simplicity and cost reduction; however, their clinical validation is currently limited.

Scaling up exoskeleton deployment in LMICs remains unrealistic with current pricing, infrastructure, and support requirements. This highlights the need for research and policy focused not only on technological advancement but also on cost-effective, accessible, and locally manufacturable solutions. In addition, further evaluation of device durability, availability of technical support, and reimbursement frameworks is an essential step toward equitable access.

## 11. Artificial Intelligence integration and real-world deployment

Devices like the Biomotum Spark (Tagoe et al., 2023) and the portable pediatric knee exoskeleton (Zhang et al., 2024) show how the incorporation of artificial intelligence (AI) greatly enhance the functionality of exoskeletons. Based on gait patterns and neuromuscular signals, such as electromyography (EMG) and electroencephalography (EEG), these devices use machine learning algorithms to dynamically modify torque assistance in real-time (Tagoe et al., 2023; Zhang et al., 2024). However, there are several challenges in integrating AI for real-world use. The heterogeneity of cerebral palsy limits personalization due to small datasets, which are common in pediatric research, leading to overfitting and reducing generalizability across age groups or different levels of the Gross Motor Function Classification System.

The ethical considerations related to AI interventions encompass algorithmic biases, constraints on resources, and obstacles in communication, which present challenges to rehabilitation outcomes and exacerbate disparities in environments with limited resources (Bulea et al., 2020). Data management concerns involve General Data Protection Regulation (GDPR) compliance and protecting pediatric patients’ privacy. Implementing stringent AI validation, ethical standards, and affordable devices is crucial for safe, efficient, and fair deployment (Tibebu, n.d.; Balgude et al., 2024; Chng et al., 2025).

## 12. Advancements in training interfaces

Notable advancements have been achieved by transitioning from passive mechanical support to active and sensor-driven integration of training interfaces. Gamification is emerging as a prominent feature aimed at improving motor learning. Exoskeleton incorporates interactive elements, including virtual reality (VR) (Wallard et al., 2017, 2018, Weinberger et al., 2019, van Kammen et al., 2020) and game-based rehabilitation (Bulea et al., 2017). In contrast, earlier exoskeleton devices like Lokomat® (Wallard et al., 2017, 2018; Digiacomo et al., 2019; Weinberger et al., 2019; van Kammen et al., 2020) and MIT-Skywalker (Susko et al., 2016) relied on more rudimentary biofeedback mechanisms. The incorporation of immersive environments for practice has significantly improved patient engagement and enthusiasm, as demonstrated by the utilization of devices such as WAKE-Up Exoskeleton (Patané et al., 2017). The implementation of gamified audiovisual feedback in conjunction with powered knee exoskeletons such as PediAnklebot (Michmizos et al., 2015; Germanotta et al., 2017), ProGait (McDaid, 2017) Tethered Knee Exoskeleton (Lerner et al., 2016), and Powered Knee Exoskeleton (Lerner et al., 2016, 2017a,b; Bulea et al., 2017) has demonstrated advantages in enhancing cortical activation and fostering voluntary motor engagement. Furthermore, the interactive interface, which constitutes biofeedback mechanisms through EMG, EEG, Audiovisual feedback, and real-time torque estimation, allows the implementation of personalized rehabilitation strategies (Lerner et al., 2018, 2019a,b; Mataki et al., 2018; Gasparri et al., 2019; Ueno et al., 2019; Conner et al., 2020, 2021, 2023; Kuroda et al., 2020; Orekhov et al., 2020; Fang et al., 2021; Fang and Lerner, 2021; Harvey et al., 2021; Orekhov et al., 2021; Conner and Lerner, 2022; Fang et al., 2022; Fang and Lerner, 2022; Kuroda et al., 2022; Moll et al., 2022; Harshe et al., 2023; Kuroda et al., 2023; Moll et al., 2023; Fang and Lerner, 2024). HAL (Mataki et al., 2018; Ueno et al., 2019; Kuroda et al., 2020, 2022, 2023; Moll et al., 2022, 2023) and Lokomat (Wallard et al., 2017, 2018; Weinberger et al., 2019; van Kammen et al., 2020) incorporate VR and CVC to refine proprioceptive training.

## 13. Changes in clinical studies

The design of clinical studies involving exoskeletons has progressed from initial pilot testing to larger-scale clinical trials and feasibility assessments. Initial studies, such as the exoskeleton brake unit (Yamada et al., 2018), assessed the stability of the knee on a single-patient basis, with limited outcome measures. The preliminary investigations were limited in scale and focused on evaluating safety and basic functionality, for example, the initial pilot studies for the PediAnklebot (Michmizos et al., 2015; Germanotta et al., 2017), powered knee exoskeleton (Lerner et al., 2016, 2017a,b; Bulea et al., 2017), and untethered ankle exoskeleton (Gasparri et al., 2018; Orekhov et al., 2021; Conner and Lerner, 2022; Fang and Lerner, 2022; Conner et al., 2023; Harshe et al., 2023; Fang and Lerner, 2024). Feasibility assessments were performed for CP Walker (Bayón et al., 2016a,b), WAKE-Up Exoskeleton (Patané et al., 2017), and HAL (Mataki et al., 2018; Ueno et al., 2019; Kuroda et al., 2020, 2022, 2023; Moll et al., 2022, 2023), whereas extensive clinical trials were carried out for Lokomat® Pediatric (Wallard et al., 2017, 2018; Weinberger et al., 2019; van Kammen et al., 2020), Angel Legs (Kim et al., 2021), and HWA (Kawasaki et al., 2020). Extensive RCTs have recently been undertaken, incorporating control groups and longitudinal measurements for Lokomat® Pediatric (Wallard et al., 2017, 2018; Digiacomo et al., 2019; Weinberger et al., 2019; van Kammen et al., 2020), CP Walker (Bayón et al., 2016a,b), HAL (Mataki et al., 2018; Ueno et al., 2019; Kuroda et al., 2020, 2022, 2023; Moll et al., 2022, 2023), Angel Legs (Kim et al., 2021), and HWA (Kawasaki et al., 2020). Larger-scale multicenter studies are essential to improve the generalizability of the findings. The inclusion of standardized assessment outcome measures, including the GMFCS, Physiological Cost Index, and Six-Minute Walk Test (6MWT), has enabled a more objective evaluation of effectiveness (Bayón et al., 2016a,b; Lerner et al., 2016; Susko et al., 2016; Bulea et al., 2017; McDaid, 2017; Wallard et al., 2017, 2018; Lerner et al., 2018, 2019a,b; Mataki et al., 2018; Gasparri et al., 2019; Ueno et al., 2019; Weinberger et al., 2019; Bulea et al., 2020, 2022; Conner et al., 2020a,b, 2021, 2023; Kuroda et al., 2020, 2023; Orekhov et al., 2020; van Kammen et al., 2020; Chen et al., 2021; Delgado et al., 2021; Fang et al., 2021; Fang and Lerner, 2021, 2024; Harvey et al., 2021; Kim et al., 2021; Orekhov et al., 2021; Conner and Lerner, 2022; Fang et al., 2022; Fang and Lerner, 2022; Kuroda et al., 2022; Moll et al., 2022, 2023; Harshe et al., 2023; Tagoe et al., 2023; Zhang et al., 2024).

## 14. Improvement in outcomes over time

The advancement of exoskeleton technology has correspondingly improved clinical outcomes. Initial investigations concentrated on feasibility, revealing only limited enhancements in mobility and muscle activation. Nonetheless, advancements in more sophisticated control algorithms, lightweight materials, and adaptive training environments have led to notable enhancements in rehabilitation outcomes.

### 14.1. Knee exoskeletons

Devices including (e.g., tethered knee exoskeleton (Lerner et al., 2016), powered knee exoskeleton (Lerner et al., 2016; Bulea et al., 2017; Lerner et al., 2017a,b), PREX (Bulea et al., 2020, 2022; Chen et al., 2021) – Early prototypes primarily offered basic knee extension support, but newer versions feature adaptive resistance and real-time control, which have led to improved gait kinematics and reduced energy consumption.

### 14.2. Ankle exoskeletons

Ankle devices such as the PediAnklebot (Michmizos et al., 2015; Germanotta et al., 2017) (a robotic ankle trainer), untethered ankle exoskeleton (Gasparri et al., 2018; Orekhov et al., 2021; Conner and Lerner, 2022; Fang and Lerner, 2022; Conner et al., 2023; Harshe et al., 2023; Fang and Lerner, 2024) (a lightweight, portable device for ankle assistance), and Biomotum Spark (Tagoe et al., 2023) (an ankle exoskeleton with biofeedback and torque control) have evolved from initial designs emphasizing passive movement assistance to more advanced iterations incorporating active torque control and biofeedback mechanisms. These advancements contribute to improved muscle recruitment and enhanced step symmetry during locomotion.

### 14.3. Gait exoskeletons

Devices targeting gait (e.g., Lokomat® Pediatric (Wallard et al., 2017, 2018; Digiacomo et al., 2019; Weinberger et al., 2019; van Kammen et al., 2020), HAL (Mataki et al., 2018; Ueno et al., 2019; Kuroda et al., 2020, 2022, 2023; Moll et al., 2022, 2023), CP Walker (Bayón et al., 2016a,b), Angel Legs (Kim et al., 2021), HWA (Kawasaki et al., 2020) were originally designed for treadmill training. These have now been modified for overground walking in the real world, with demonstrated improved walking distance, posture, propulsion, and long-term motor retention in children with CP. Despite these advancements, barriers continue to exist to show sustained functional independence and guarantee that improvements are seen beyond the duration of the training.

## 15. Trends and existing gaps

Several trends were observed in the context of research on the lower-limb exoskeleton for CP. New research in these devices aims at portability and reduced weight, as seen in the untethered exoskeleton, including Biomotum Spark (Tagoe et al., 2023), HWA (Kawasaki et al., 2020), and untethered ankle exoskeletons (Gasparri et al., 2018; Orekhov et al., 2021; Conner and Lerner, 2022; Fang and Lerner, 2022, 2024; Conner et al., 2023; Harshe et al., 2023) for real-world settings. The implementation of AI-driven adaptive control systems is being integrated into many devices, including the CP Walker (Bayón et al., 2016a,b), WAKE-Up Exoskeleton (Patané et al., 2017), and HAL (Mataki et al., 2018; Ueno et al., 2019; Kuroda et al., 2020, 2022, 2023; Moll et al., 2022, 2023), permitting real-time gait modifications to enhance the experience of patients and improve motor learning. Similarly, another trend is to incorporate multisensory integration, advanced haptic, and neurofeedback to improve the effectiveness of training and engagement of patients during interventions. Devices such as the WAKE-Up Exoskeleton (Patané et al., 2017) have successfully provided leverage toward these interactive features to improve rehabilitation outcomes.

Despite the evidence of short-term benefits, there is still a significant lack of knowledge regarding the long-term efficacy of exoskeleton application in CP rehabilitation. To address the diversity of neuromotor impairments among populations impacted by CP, device customization is essential, as demonstrated in HAL (Mataki et al., 2018; Ueno et al., 2019; Kuroda et al., 2020, 2022, 2023; Moll et al., 2022, 2023) and CP Walker (Bayón et al., 2016a,b).

## 16. Future directions

The new technologies in exoskeletons should be focused on several major developments. Of particular significance is cable-driven actuation, which can be used to minimize the overall weight of exoskeletons, while still interacting with the human subjects, for example, cable-driven active leg exoskeleton (C-ALEX) (Hidayah et al., 2020), tethered pelvic assist device (TPAD) (Kang et al., 2017), mobile tethered pelvic assist device (mTPAD) (Martelli et al., 2017), and robotic upright stand trainer (RobUST) (Rejc et al., 2024). Technologies such as the WAKE-Up Exoskeleton (Patané et al., 2017) and Angel Legs (Kim et al., 2021) are in the process of integrating such mechanisms. Of particular interest is the implementation of AI-based assistance, wherein personalized machine learning methods for gait optimization can make such devices more flexible for each individual. Moreover, cost-effective production techniques are being studied to make exoskeletons available to rehabilitation centers, thereby making them more marketable.

There is also greater interest in making the user more comfortable with more advanced control schemes so that exoskeletons can provide more intuitive assistance based on real-time feedback from the user. This requires hybrid actuation schemes that combine active and passive elements to make the exoskeleton more energy-efficient and less exhausting for the user. There is also likely to be advanced material science that makes exoskeletons stronger, more flexible, and evenly weight-bearing so that they are more comfortable and usable for longer periods.

Eventually, future technologies should be focused on expanding devices to reach more people, including powered knee exoskeletons (Lerner et al., 2016, 2017a,b; Bulea et al., 2017), untethered exoskeletons (Gasparri et al., 2018;Orekhov et al., 2021; Conner and Lerner, 2022; Fang and Lerner, 2022, 2024; Conner et al., 2023; Harshe et al., 2023) at the ankles, passive leg exoskeletons for CP, and whole-body rehabilitation devices like the HAL (Mataki et al., 2018; Ueno et al., 2019; Kuroda et al., 2020, 2022, 2023; Moll et al., 2022, 2023). They will be more personalized, effective, and available to more people with neuromotor impairment.

Importantly, future efforts must also address the affordability and accessibility of these technologies in LMICs, where the prevalence of CP is often higher and resources are limited (Kakooza-Mwesige et al., 2017).

Overall, exoskeleton technology has advanced greatly, but further research needs to be conducted to make these usable, effective in real-world use, and accepted in clinical and home settings.

## 17. Conclusion

This systematic review provides a comprehensive assessment of lower limb exoskeletons used in the rehabilitation of individuals with CP. The findings point to a number of practical conclusions and research recommendations: (i) Design Evolution: There has been a shift from rigid, tethered models to lightweight, modular, and portable exoskeletons designed for real-world applications. The integration of adaptive control, biofeedback, and gamified training has enhanced therapeutic outcomes and increased user engagement. (ii) Control and Feedback Integration: There is a growing use of advanced control strategies, including assist-as-needed, impedance-based, and machine learning-adaptive controls, which enhance device responsiveness and personalization. Biofeedback mechanisms, particularly real-time gait phase detection, further promote neuromuscular engagement. (iii) Clinical Benefits and Gaps: Studies consistently report improvements in gait parameters (e.g., speed, stride length, joint ROM) and energy efficiency. However, clinical validation remains limited in terms of sample size, study duration, and long-term follow-up. (iv) Future Research Directives: Conduct large-scale, long-term clinical trials to assess sustained outcomes, develop cost-effective, scalable exoskeletons for low-resource settings, incorporate AI and wearable sensor systems to enable personalized therapy, and standardize outcome measures for comparison across studies. In conclusion, exoskeletons hold significant promise for pediatric CP rehabilitation. Continued interdisciplinary collaboration is essential for translating these innovations into accessible, effective clinical solutions.

## Data Availability

All data and materials presented in this systematic review are derived from peer-reviewed scientific publications.
